# Urinary Retention, Incontinence, and Dysregulation of Muscarinic Receptors in Male Mice Lacking *Mras*

**DOI:** 10.1371/journal.pone.0141493

**Published:** 2015-10-30

**Authors:** Annette Ehrhardt, Bin Wang, Andrew C. Yung, Yanni Wang, Piotr Kozlowski, Cornelis van Breemen, John W. Schrader

**Affiliations:** 1 The Biomedical Research Centre, The University of British Columbia, 2222 Health Sciences Mall, Vancouver, British Columbia, Canada; 2 The University of British Columbia MRI Research Centre, Life Sciences Centre, 2350 Health Sciences Mall, Vancouver, British Columbia, Canada; 3 The University of British Columbia, Departments of Radiology and Urologic Sciences, 818 West 10th Ave., Vancouver, British Columbia, Canada; 4 The University of British Columbia, Department of Pharmacology and Therapeutics, 2176 Health Sciences Mall, Vancouver, British Columbia, Canada; Texas A&M University Health Science Center, UNITED STATES

## Abstract

Here we show that male, but not female mice lacking expression of the GTPase M-Ras developed urinary retention with distention of the bladder that exacerbated with age but occurred in the absence of obvious anatomical outlet obstruction. There were changes in detrusor morphology in *Mras* ^*-/-*^ males: Smooth muscle tissue, which exhibited a compact organization in WT mice, appeared disorganized and became increasingly ‘layered’ with age in *Mras* ^*-/-*^ males, but was not fibrotic. Bladder tissue near the apex of bladders of *Mras* ^*-/-*^ males exhibited hypercontractility in response to the cholinergic agonist carbachol in *in vitro*, while responses in *Mras* ^*-/-*^ females were normal. In addition, spontaneous phasic contractions of detrusors from *Mras* ^*-/-*^ males were increased, and *Mras* ^*-/-*^ males exhibited urinary incontinence. We found that expression of the muscarinic M2 and M3 receptors that mediate the cholinergic contractile stimuli of the detrusor muscle was dysregulated in both *Mras* ^*-/-*^ males and females, although only males exhibited a urinary phenotype. Elevated expression of M2R in young males lacking M-Ras and failure to upregulate M3R with age resulted in significantly lower ratios of M3R/M2R expression that correlated with the bladder abnormalities. Our data suggests that M-Ras and M3R are functionally linked and that M-Ras is an important regulator of male bladder control in mice. Our observations also support the notion that bladder control is sexually dimorphic and is regulated through mechanisms that are largely independent of acetylcholine signaling in female mice.

## Introduction

Micturition, the storage and periodic elimination of urine, depends on the coordinated actions of detrusor and sphincter musculature in the lower urinary tract. Complex neural systems in the brain, spinal cord and peripheral ganglia regulate the coordination between the organs involved (bladder, urethra, and urethral sphincter), which allows for voiding the bladder at appropriate times (reviewed in [1]). During the normal storage phase, sympathetic nerves (e.g. the Hypogastric nerve) release noradrenaline, which acts to relax the detrusor by binding to β_3_-adrenergic receptors, and to contract urethral smooth muscle by activating α_1_-adrenergic receptors [2]. In addition, the somatic Pudenal nerve releases acetylcholine (ACh) to contract the skeletal muscle tissue of the external sphincter. When safe to void, ACh is released in response to parasympathetic signals from the Pelvic nerve, which stimulates muscarinic receptors to cause contraction of smooth muscle fibers in the detrusor. Muscarinic M3 receptors (M3R) are thought to be the main receptors to initiate detrusor contractions, with M2R also playing a role [3–6]. Parasympathetic purinergic signaling via P2X receptors also supports the initiation of voiding. While contributions appear to be small under healthy conditions, they can increase in disease [7].

Disorders of micturition can encompass complications with the process of voiding, leading to urine retention, or with urine storage, causing leakage or incontinence. Incontinence is associated with a variety of underlying problems, including congenital or idiopathic abnormalities of the urinary system (e.g. in idiopathic detrusor overactivity), neurologic conditions resulting in detrusor overactivity (such as multiple sclerosis or Parkinson’s disease), or inflammation, as in cystitis [8–11]. Urinary retention is often caused by outflow obstruction due to anatomic abnormalities (e.g. prostate enlargement or neoplasia, or uroliths), or by neurological disorders (e.g. upper and lower motor neuron lesions). Retention may paradoxically be accompanied by leakage, and may lead to enlargement of the bladder. This condition frequently develops in older men, often as a result of prostate disorders [12–15].

Although a role for Ras proteins in bladder function has not been described, we unexpectedly discovered that male, but not female mice lacking expression of the GTPase M-Ras/R-Ras3 frequently presented with enlarged urinary bladders. M-Ras belongs to the Ras family of proteins that comprises well over 150 members. It is very closely related to the ‘classical’ p21 Ras proteins (H-Ras, N-Ras, K-Ras) that have established roles in human cancer [16]. Although M-Ras can behave like an oncogene and transform many cell types *in vitro* [17–22], its role in human cancer is presently unclear. Some tumors exhibit overexpression of *MRAS* [23], but no mutations in oncogenic hot spots have been reported in well over 20,000 sequenced cancers (Sanger COSMIC database). Based on our initial observation of urinary retention, we performed a series of experiments to characterize the bladder phenotype, and found that *Mras* ^*-/-*^ males also exhibited incontinence and hypercontractility of bladder tissue. To identify potential underlying molecular causes of the bladder phenotype, we analyzed expression levels of M2 and M3 muscarinic receptors and found that these were dysregulated in *Mras* ^*-/-*^ mice. Given their importance in the regulation of bladder contractions and voiding, this result suggests that altered expression levels of M2R and M3R likely contributed to the changes in bladder control in *Mras* ^*-/-*^ males.

## Methods

### Animals

*Mras* ^*-/-*^ (KO) mice were obtained via contract from Lexicon Genetics and were backcrossed to a C57Bl/6 background to F10. Wild-type (WT) C57Bl/6 and *Mras* ^*-/-*^ mice were housed in a controlled environment with 12 hour light/dark cycles and access to water and standard rodent chow ad libitum. For bladder magnetic resonance imaging (MRI) studies, live mice (27–35 weeks old) were sedated by isoflurane inhalation and imaged with a 2.35 Tesla SMIS/Bruker MRI system. A series of T1-weighted images were acquired using a spin echo pulse sequence (echo time = 10 ms, repetition time = 500 ms, field of view = 4 cm, 128x128 pixels, slice thickness = 2 mm). The T1-weighting of the images provide good contrast between the bladder water and the surrounding tissue. Separate image sets were collected in the axial, sagittal and coronal orientations. Bladder volumes calculated from these three orientations were averaged together for each mouse in order to improve precision of the measurement. Between one and five measurements (on different days and averaged) were obtained per mouse. For urine collection, urination was stimulated from live mice by having a person unfamiliar to them place them on a wire cage lid and gently holding them by their bodies and tails as they tried to move forward. We estimated the average water intake per mouse by weighing water bottles of group-housed mice (2–5 mice of the same age and sex per cage) before and after five days of housing. We strictly followed general guidelines set forth by the Canadian Council on Animal Care. Animal protocols were approved by the Animal Care Committee of the University of British Columbia (Protocol Numbers: A08-0202 and A13-0213).

### Urine and bladder tissue analysis

Protein concentration of mouse urine was measured using bicinchoninic acid assay (BCA) reagents (Pierce). Urinary creatinine was measured using a colorimetric kit (Enzo Life Sciences). Masson's Trichrome or H&E was used to stain five μm histological sections of bladder tissue (performed by WaxIt Histology, Inc.). The Sirius Red Collagen Detection Kit (Chondrex) was used to quantitate collagen in bladder tissue from mice that were 3–3.5 months old. Scoring of the degree of detrusor smooth muscle layering: We estimated the degree of layering by roughly categorizing randomly photographed H&E-stained bladder sections. A score of 0 was given to a normal, compact appearance. Mild to medium layering with some white spaces between smooth muscle layers was scored as 1, and severe layering with a “bacon-like” appearance was scored 2 (S1A Fig).

### Bladder contractility

Two tissue rings perpendicular to the sphincter-apex axis, approximately 1.5–2 mm wide, were cut from freshly isolated bladders. The tissue rings were mounted for *in vitro* force registration on a wire myograph (Danish Myo Technology, Aarhus, Denmark, Model 610 M). The mounted tissue was bathed in physiological salt solution (PSS; 119 mM NaCl, 4.7 mM KCl, 1.18 mM KH_2_PO_4_, 24 mM NaHCO_3_, 1.17 mM MgSO_4_ · 7H_2_O, 1.6 mM CaCl_2_, 5.5 mM glucose, 26 μM EDTA) at 37°C for one hour during which resting tension was obtained. To obtain the maximal possible contraction force of each tissue ring we first added KCl (60 mM K^+^ iso-osmotic substitution for Na^+^). After washing out KCl with PSS we added increasing concentrations of carbachol (Sigma). Each dose was followed by a wash with PSS before addition of the next highest concentration. Bladder smooth muscle contraction is expressed as stress (in mN per mm^2^ cross-section). Since all tissues were treated equally and cut into strips of 1.5–2 mm widths, and since there was also no difference in the thickness of the detrusors between WT and *Mras* ^*-/-*^ males (S1B Fig), it can be assumed that the force generated (in mN) reflects the stress generated (in mN/mm^2^) by the bladder tissues.

### Urine scent marking tests

Mice were each placed in a clean cage that was lined with filter paper and were allowed to mark their new territory with urine spots for 15 minutes. The filter paper was photographed under UV light to visualize urine spots, and the number of spots was enumerated. Males in this test were 5–7 months old, females were 10–12 months old.

### Quantitative RT-PCR

To determine expression levels of M2R and M3R (muscarinic receptors 2 and 3; *Chrm2* and *Chrm3*) RNA was extracted from bladders with Trizol (Invitrogen) and reverse transcribed with Superscript (Invitrogen). We used the following primers for real-time PCR on an ABI HT7900 instrument (Applied Biosystems). M2R forward: CCAGCCAGACTCCACCAGAT. M2R reverse: GTTCAGTAGTCAAGTGGCCAAAGA. M3R forward: GTGGACTGTGGATTGAGTGAACC. M3R reverse: GTCACTTGGTCAGAACGCAGC. RP2 (RNA polymerase II; *PolR2A*) was used as a reference gene. RP2 forward: GTCTTCCTGCGATGCATTGA. RP2 reverse: AGTGCATGTACACCTTGCTGATC.

### Data analysis

Statistical analyses were carried out using Graphpad Prism 6.0. Significance was considered at *p*<0.05; *p<0.05, **p<0.01, ***p<0.001, ****p<0.0001.

## Results

### Enlarged bladders and urinary retention in *Mras* ^*-/-*^ males

**Fig 1:**
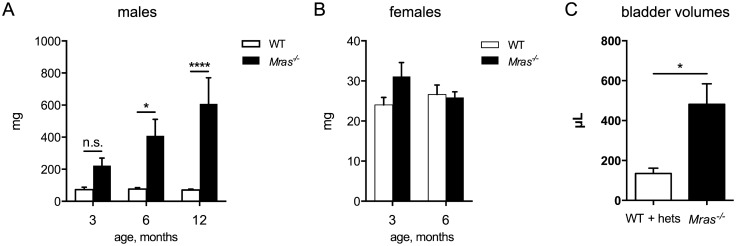
Enlarged bladders in *Mras* ^*-/-*^ males. (A) Postmortem full bladder weights of male mice at different ages. Number of bladders tested: WT males: n = 15, three months; n = 17, six months; n = 21, 12 months. *Mras* ^*-/-*^ males: n = 13, three months; n = 11, six months; n = 12, 12 months. Two-way ANOVA: There was a significant effect of genotype, F_1,83_ = 39.93, *p*<0.0001. However, a significant interaction between genotype and age was detected (*p* = 0.0142). The differences in bladder weights were significant at six months (*p* = 0.0104) and at 12 months (*p*<0.0001). (B) Postmortem full bladder weights of female mice at three and six months of age. WT females: n = 8, three months; n = 7, six months. *Mras* ^*-/-*^ females: n = 9 for both three and six months. Two-way ANOVA: not significant. (C) Bladder volumes of live males were determined by MRI; t-test: *p* = 0.0146; WT+hets, n = 7; *Mras* ^*-/-*^, n = 10. Error bars in (A, B, C) represent SEM.

During early inspections and dissections of mice lacking M-Ras we noted that, while all other organs appeared normal, the urinary bladders of *Mras* ^*-/-*^ males were frequently enlarged. Postmortem full bladder weights were significantly higher in six and 12 month-old *Mras* ^*-/-*^ males compared to WT males (*p* = 0.0104 and *p*<0.0001 at six and 12 months, respectively), and the difference increased with age (Fig 1A). In 12 month-old *Mras* ^*-/-*^ males bladders were grossly distended and about six times larger than those of WT males. In contrast, bladders from *Mras* ^*-/-*^ females were small and similar in weight to WT female bladders (Fig 1B). MRI analysis showed that urinary bladders of *Mras* ^*-/-*^ males were also significantly larger in volume in live mice (*p* = 0.0146; Fig 1C).

### Layered appearance of detrusor smooth muscle in *Mras* ^*-/-*^ bladders

**Fig 2:**
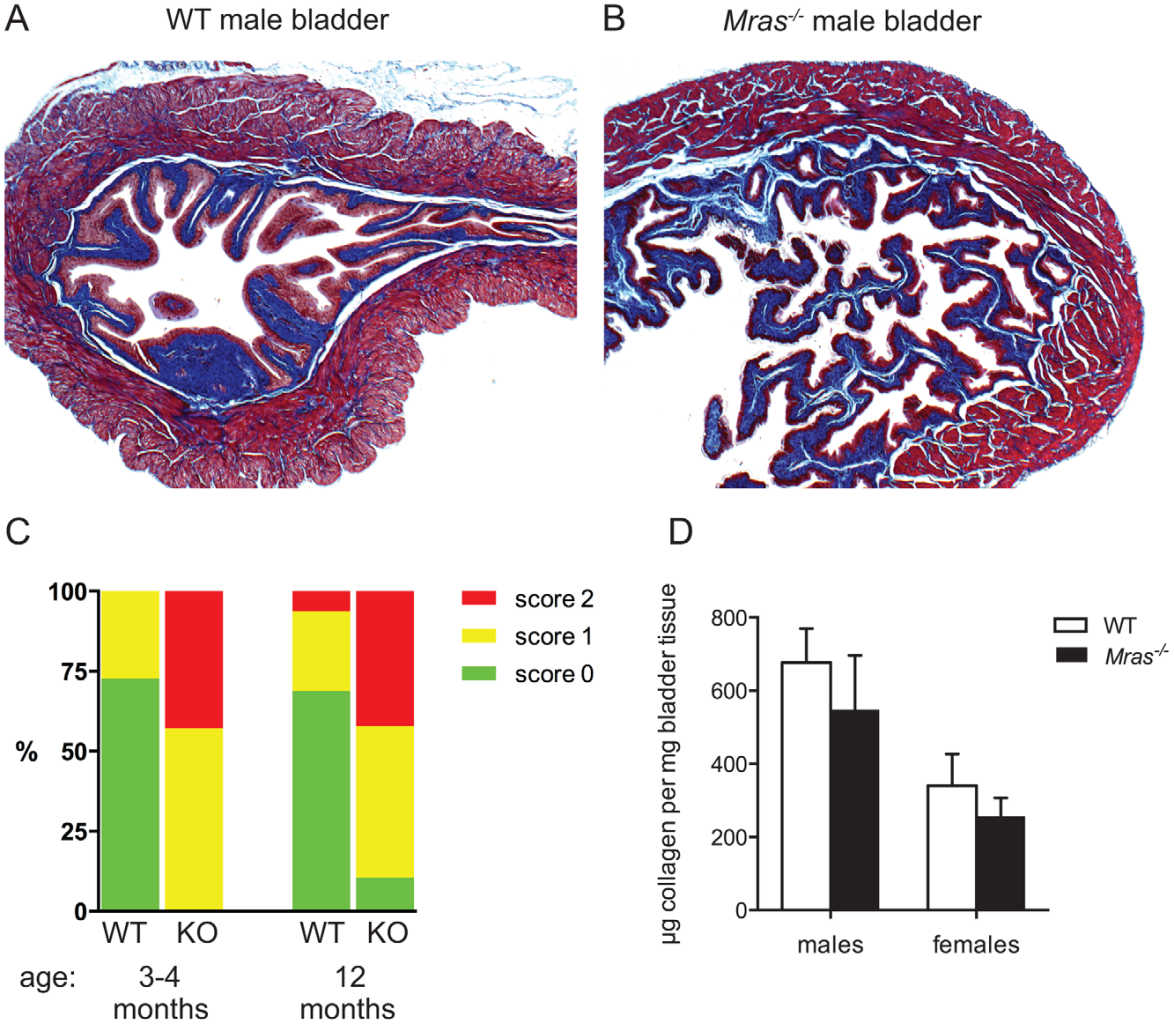
Increased layering of detrusor smooth muscle tissue in the absence of fibrosis in *Mras* ^*-/-*^ males. (A, B) Histological sections of a WT (A) or *Mras* ^*-/-*^ (B) male bladder were stained with Masson’s Trichrome to visualize collagen in blue, muscle in red, and nuclei in purple. 5X magnification. (C) Random histological sections of bladder tissue from WT and *Mras* ^*-/-*^ males at 3–4 months or 12 months of age were visually inspected and scored for the degree of disorganization, or ‘layered’ appearance, of detrusor smooth muscle. (D) Quantification of collagen in bladders of WT and *Mras* ^*-/-*^ mice. Two-way ANOVA: There was a significant effect of gender, F_1,17_ = 51.59, p<0.0001, but there were no significant differences between WT vs. *Mras* ^*-/-*^ male or WT vs. *Mras* ^*-/-*^ female samples. Error bars represent SD, n = 5 for WT males and *Mras* ^*-/-*^ males and females, n = 6 for WT females.

We hypothesized that with increased bladder volumes in *Mras* ^*-/-*^ males there could be changes in bladder morphology. We did not observe differences in the thickness of the detrusor (S1B Fig), but there was an increased amount of disorganization, or layering, of the internal longitudinal, outer circular, and outermost longitudinal smooth muscle layers of detrusors from *Mras* ^*-/-*^ males (Fig 2A–2C and S1A Fig). Bladders from *Mras* ^*-/-*^ males rarely showed normal, compact organization of smooth muscle tissue. Instead, 42% of bladders from both three and 12 month-old *Mras* ^*-/-*^ males showed severe layering, while only 6% of bladders from 12 month-old WT males exhibited severe layering and none in three month-old WT males (Fig 2C). Staining with Masson's Trichrome revealed that the increased layering and ‘bacon-like’ appearance of smooth muscle tissue was not due to fibrosis (Fig 2A and 2B). We further determined that the amount of collagen in *Mras* ^*-/-*^ bladders was equivalent to that contained in WT bladders both in male and female mice (Fig 2D).

### Increased protein concentration

**Fig 3:**
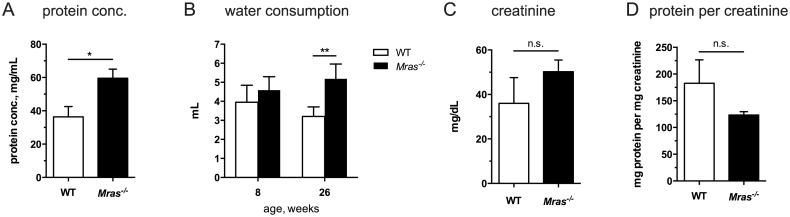
Elevated urinary protein concentration in *Mras* ^*-/-*^ males. (A) The protein concentration of randomly collected urine samples was elevated in *Mras* ^*-/-*^ males; t-test: *p* = 0.0115. (B) Water consumption by young (8 week-old) and older (26 week-old) males. Two-way ANOVA: There was a significant effect of genotype, F_1,20_ = 18.23, *p* = 0.0004, but a significant interaction between genotype and age was also detected (*p* = 0.0407). The difference in water consumption was significant at 26 weeks (*p* = 0.0011). Eight week-old mice: WT and *Mras* ^*-/-*^, n = 6; 26 week-old mice: WT, n = 5; *Mras* ^*-/-*^, n = 7. Numbers represent cages of group-housed mice but water consumption was calculated on average per mouse. Error bars represent SD. (C) Creatinine levels were similar in WT and *Mras* ^*-/-*^ males; t-test: not significant. (D) Normalized to creatinine, protein content in *Mras* ^*-/-*^ urine was not significantly different from that of WT urine; (A, C, D) WT, n = 9; *Mras* ^*-/-*^, n = 13; error bars represent SEM.

We next analyzed urine samples from WT and *Mras* ^*-/-*^ mice. Protein content in randomly collected samples was significantly higher in urine from *Mras* ^*-/-*^ males (*p* = 0.0115; Fig 3A). The higher protein concentration may have been due to decreased water intake. However, young (three month-old) *Mras* ^*-/-*^ males drank similar amounts of water as WT males, and water intake in old (12 month-old) *Mras* ^*-/-*^ males was actually increased (*p* = 0.0011; Fig 3B). However, urinary creatinine levels, although variable, were similar in WT and *Mras* ^*-/-*^ males (Fig 3C), and when protein concentration was normalized to creatinine in each urine sample we noted that values were similar in WT and *Mras* ^*-/-*^ males (Fig 3D). These results indicated that kidney function s likely normal in *Mras* ^*-/-*^ mice and that urine may be normally produced but retained in the bladders of *Mras* ^*-/-*^ males.

Urine retention could be the consequence of an anatomical obstruction in or near the urethra or sphincter. This can be caused by an enlarged prostate gland, for example. However, there were no visible differences in size or appearance between the prostate glands of WT and *Mras* ^*-/-*^ males (not shown). In addition, *Mras* ^*-/-*^ males readily urinated when picked up by an unfamiliar experimenter, or when placed into a fresh cage lined with filter paper to mark new territory (S2A and S2B Fig). Kidney weights were also similar in WT and *Mras* ^*-/-*^ males (S2C Fig). This may indicate an absence of hydronephrosis that can accompany urinary retention with outflow obstruction. Thus, we concluded that anatomical obstruction was likely not the cause of urinary retention.

### Detrusor hypercontractility in *Mras* ^*-/-*^ males

**Fig 4:**
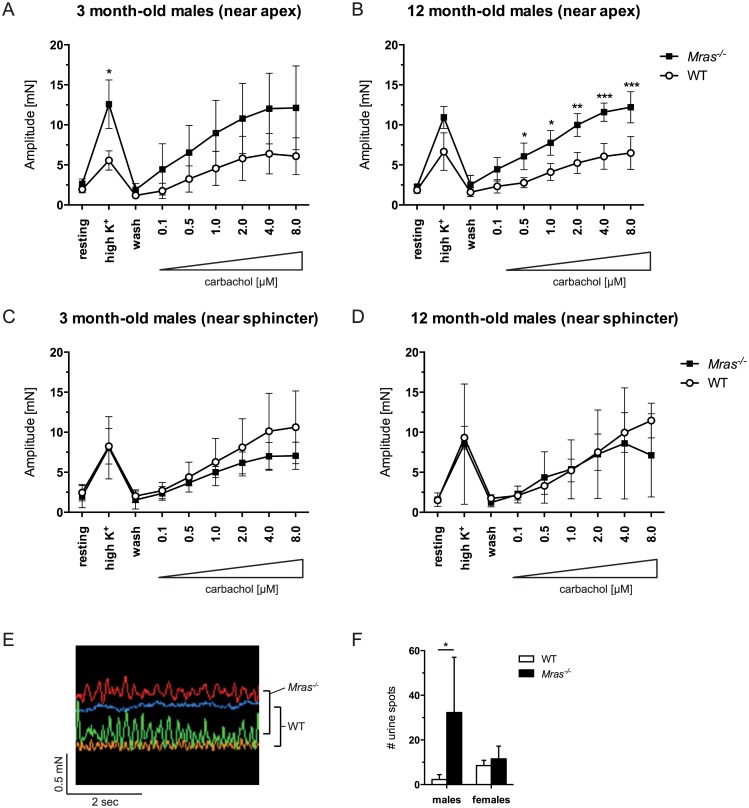
Detrusor hypercontractility, instability, and incontinence in *Mras* ^*-/-*^ males. (A-D Bladder contractility was measured in two regions of WT and *Mras* ^*-/-*^ male bladders at three and 12 months of age, near the apex (A, B) and near the sphincter (C, D). Error bars represent SDs; n = 4 for three month-old males, n = 3 for 12 month-old males. Two-way ANOVA with Bonferroni’s *post hoc* tests: Apex tissue, three month-old males: Significant effect of genotype (F_1,42_ = 23.28, *p*<0.0001). Tissue near sphincter, three month-old males: Significant effect of genotype (F_1,42_ = 6.557, *p* = 0.0141). Apex tissue, 12 month-old males: Significant effect of genotype (F_1,28_ = 75.81, *p*<0.0001). Tissue near sphincter, 12 month-old males: No significant effect of genotype. No significant interactions between genotype and carbachol concentration were detected except for apex samples from 12 month-old males (*p* = 0.0499). (E) Increased spontaneous bladder contractions (oscillations) in the absence of stimulation in bladder tissue rings from *Mras* ^*-/-*^ males. Two WT traces are shown in yellow and blue, two *Mras* ^*-/-*^ traces are shown in red and green. (F) Urine scent marking test: Number of urine spots produced in a clean cage by WT and *Mras* ^*-/-*^ mice over a period of 15 minutes. Two-way ANOVA with Bonferroni’s *post hoc* analysis: There was a significant effect of genotype (F_1,12_ = 6.635, *p* = 0.0243) with significantly more urine spots produced by *Mras* ^*-/-*^ males compared to WT males (*p* = 0.0124), while *Mras* ^*-/-*^ and WT females produced similar numbers of spots.

We next investigated if there were defects in the neurological circuitry responsible for signaling to void the bladder in *Mras* ^*-/-*^ males. The parasympathetic nervous system controls detrusor muscle contraction mainly by cholinergic signals acting on muscarinic M3 and M2 receptors. We used the cholinergic agonist carbachol to stimulate contractions of isolated tissue from two areas of the bladder, close to the apex and close to the sphincter. Responses in female WT and *Mras* ^*-/-*^ bladders were similar in both regions of the bladders (S3 Fig). However, there were differences in the responses to carbachol in male bladder tissues. When bladder tissue close to the apex of the bladder was tested, significant differences in contractility were observed between WT and *Mras* ^*-/-*^ tissues both in young (three month-old) males and in old (12 month-old) males (*p*<0.0001 in both cases; Fig 4A and 4B). The differences in the magnitudes of the responses were significant in bladders of 12 month-old males but not in three month-old males (Fig 4A and 4B). Carbachol responses in bladder tissue closer to the sphincter of the bladder were similar in WT and *Mras* ^*-/-*^ males both at three months and at 12 months of age (Fig 4C and 4D). There was a significant effect of genotype in bladder tissues from three month-old males (*p* = 0.0141), but no significant differences between individual values at any specific carbachol concentrations were detected. Interestingly, bladder tissue that was close to the apex of the bladder of *Mras* ^*-/-*^ males also showed hyperresponsiveness to K^+^ depolarization compared to WT male bladder tissue both at three and 12 months of age (Fig 4A and 4B). However, bladder tissue that was closer to the sphincter did not show differences in contractility between young or old WT and *Mras* ^*-/-*^ males in response to K^+^ (Fig 4C and 4D).

### Detrusor instability and incontinence in *Mras* ^*-/-*^ males

We also noted that bladder tissue rings from *Mras* ^*-/-*^ males exhibited increased amplitudes of spontaneous bladder contractions (SBCs) under resting, unstimulated conditions (Fig 4E). The enhanced basal detrusor contractility diminished after K^+^ and subsequent carbachol stimuli (not shown). Retention, hypercontractility and instability (increased SBCs) often coincide with urinary incontinence. Indeed, *Mras* ^*-/-*^ males produced significantly more urine spots during a scent marking test than WT males (*p* = 0.0124; Fig 4F), which indicated that *Mras* ^*-/-*^ males exhibited incontinence. WT and *Mras* ^*-/-*^ females produced similar numbers of spots in this test, suggesting normal bladder function (Fig 4F).

### Muscarinic receptor expression

**Fig 5:**
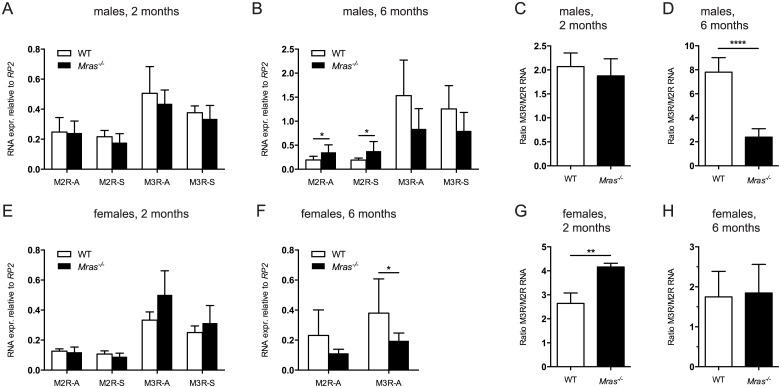
M2R and M3R expression levels in male and female WT and *Mras* ^*-/-*^ mice over time. (A, B, E, F) RNA levels of M2R and M3R in two and six month-old males (A, B) and females (E, F) in bladder tissue near the apex (suffix A) and near the sphincter (suffix S). (C, D, G, H) The ratios of M3R to M2R levels in individual bladders were determined in bladder tissue from two and six month-old WT and *Mras* ^*-/-*^ males (C, D) and females (G, H). Error bars represent SD; n = 4 for two month-old males; n = 6 or n = 7 for six month old WT or *Mras* ^*-/-*^ males, respectively; n = 3 for two month-old females, n = 4 or n = 8 for six month-old WT or *Mras* ^*-/-*^ females, respectively. Statistical analyses were performed using t-tests. Six month-old males: *p* = 0.0435 and *p* = 0.0450 for M2R-A and M2R-S (B), respectively; *p*<0.0001 for the ratio of M3R/M2R (D). Six month-old females: *p* = 0.0457 for M3R-A (F). Two month-old females: *p* = 0.0045 for the ratio of M3R/M2R (G).

It was previously reported that the concentration of muscarinic receptors may be higher near the apex of rat urinary bladders [24], the area where we observed exaggerated contractile responses to carbachol and K^+^ in bladders of old *Mras* ^*-/-*^ males. We tested mRNA expression levels of M2R and M3R in WT and *Mras* ^*-/-*^ bladders both near the sphincter and the apex of bladders by quantitative RT-PCR. Transcript levels of M2R and M3R were very similar in these two regions of the bladder both in two month-old mice (males or females; Fig 5A and 5E) and in six month-old mice (males or females; Fig 5B and 5F). However, by six months of age *Mras* ^*-/-*^ males expressed significantly more M2R than WT males (*p* = 0.0435 and *p* = 0.0450, tissue near apex or sphincter, respectively) while levels of M3R were similar (Fig 5B). In contrast, six month-old female mice lacking *Mras* expressed significantly less M3R compared to their WT counterparts (*p* = 0.0457; Fig 5F).

When we determined the expression levels of M3R and M2R relative to each other in individual bladders we observed striking differences between WT and *Mras* ^*-/-*^ mice. These ratios changed over time in both male and female mice. While the M3R/M2R ratio increased about four-fold in WT males between two and six months of age, it did not change in *Mras* ^*-/-*^ males (*p*<0.0001; Fig 5C and 5D). In contrast, the relative expression levels of M3R to M2R did not change over time in WT females, but decreased by about half in *Mras* ^*-/-*^ females between two and six months of age (*p* = 0.0045; Fig 5G and 5H). Thus, the regulation of expression of the cholinergic receptors for M2 and M3 was sexually dimorphic in WT mice. Moreover, these data suggest that muscarinic receptor levels were dysregulated in both *Mras* ^*-/-*^ males and females. While this may help explain the differential contractile responses *in vitro* and urine retention *in vivo* in WT and *Mras* ^*-/-*^ males, the data also supports the notion that female mouse bladders may primarily be controlled through neurotransmitters other than ACh [6].

## Discussion

How members of the Ras family of GTPases are involved in bladder control and function is largely unexplored. Rho is activated by cholinergic stimulation and is and critical for detrusor contraction *in vitro* [25]. The activation of R-Ras and Rap2B downstream of M3R signaling has been reported. Rap2B plays a role in the activation of PLCε and calcium release from the sarcoplasmatic reticulum [26] and R-Ras may be involved in PLD activation [27]. Thus, to our knowledge M-Ras is the first known Ras protein that contributes to bladder control *in vivo*. The absence of M-Ras in mice revealed a male-specific bladder phenotype, even though muscarinic receptors were dysregulated in both males and females. *Mras* ^*-/-*^ males exhibited bladder distention, urinary retention and incontinence, and detrusors lacking M-Ras exhibited enhanced SBCs and hypercontractility. The dysregulation of M2R and M3R expression in *Mras* ^*-/-*^ mice is likely a major cause of these abnormalities, but M-Ras could affect bladder control on other levels as well.

The muscarinic receptors M3R and M2R play an important role in the control of micturition, with M3R being the main receptor to mediate detrusor contractions. Bladders from mice that are deficient in either receptor or both in combination show decreased, although not completely abolished, contractile responses to cholinergic stimuli [3, 6, 28]. Although there is no gender difference *in vitro*, the absence of M3R severely affects male but not female bladder function *in vivo*. Thus, M3R is critical for voiding in male mice, and males lacking M3R expression develop enlarged bladders [3, 6]. In contrast, the contributions of muscarinic receptors to female micturition are small, and voiding is facilitated by purinergic signaling instead [6, 7]. Our data suggests that M-Ras is important for the regulation of expression levels of M2R and M3R in both male and female mice, whereas its absence did not affect the expression levels of M1R or some of the other receptors or receptor components expressed in the bladder, such as the β1 subunit of the large conductance calcium-activated potassium channels (BK_Ca_β1), fibroblast growth factor receptor 2 (FGFR2), or Neuropilin 2 (NRP2; S5A, S5B, S5E and S5F Fig). We found that while M2R levels remain constant over time in WT males, M3R was upregulated approximately 3-4-fold between the ages of two and six months. Consequently, M3R to M2R ratios increased over time. The upregulation of M3R did not occur in *Mras* ^*-/-*^ males; instead, expression levels of M2R increased, resulting in severely skewed M3R/M2R ratios. In female mice, M2R and M3R expression levels remained fairly constant over time in WT mice. Young *Mras* ^*-/-*^ females, however, exhibited higher M3R/M2R ratios, but this was corrected by six months of age. Thus, it seems that the absence of M-Ras prevented adequate M3R expression. The bladder phenotypes in male and female mice lacking M3R or *Mras* ^*-/-*^ are very similar with respect to urinary retention, which indicates that M-Ras and muscarinic receptors may be functionally linked.

Given that M3R and M2R signal through different activating or inhibitory pathways, balancing the levels of the two receptors is likely of importance to the regulation of bladder contractions especially in male mice. M3R preferentially couples to G_q/11_ to activate phosphoinositide hydrolysis leading to calcium mobilization, which initiates contraction. Although M2R couples to inhibitory G_i/o_, detrusor contractions may be indirectly triggered by inhibiting relaxation mediated through β-adrenergic or purinergic signals [29–32]. Lower than normal M3R/M2R levels could mean that the threshold for M3R signaling for active detrusor contraction cannot be reached under normal, non-stressed conditions *in vivo*. However, since isolated male *Mras* ^*-/-*^ detrusors are hyperreactive *in vitro*, other factors must contribute. For example, bladders from old males may increasingly experience purinergic stimulation [33], which could mean a shift towards the indirect pathway to contraction via M2R with its elevated expression levels in old *Mras* ^*-/-*^ males. It has been suggested that the contribution of M2R to detrusor contraction increases in certain urological disorders, e.g. in obstructed or denerved rat bladders, or in patients with neurogenic bladder dysfunction [34–37].

Although we did not observe obvious anatomical obstruction in *Mras* ^*-/-*^ males, we cannot exclude the possibility of obstruction. Outflow obstruction can lead to hypersensitivity to ACh and detrusor overactivity, although this is not always the case [2, 38–40]. Increased sensitivity to ACh has been attributed to elevated expression levels of P2X1 receptors in humans with obstructed bladders, but other studies suggest that P2X1 receptor signaling or expression levels do not affect bladder function or modulate cholinergic responses in rodents [41–45]. Young *Mras* ^*-/-*^ males expressed P2X1R at levels that were similar to WT males, but older (six month-old) *Mras* ^*-/-*^ males expressed significantly lower levels than WT males (S5C Fig). It remains unclear if there were changes in sensitivity to ACh due to changes in P2X1R expression levels. However, the differences in muscarinic receptor expression may help explain the differential contractile responses *in vitro* and urine retention *in vivo* in WT and *Mras* ^*-/-*^ males. In addition, the data supports the notions that M3R is critical for voiding in males, and that female mouse bladders may primarily be controlled through neurotransmitters other than ACh.

The bladder smooth muscle undergoes changes in intrinsic activity during the first few weeks postnatal. Newborn rat bladders exhibit increased spontaneous phasic contractions in the absence of stimulation that are high in amplitude and low in frequency [46–48]. These spontaneous bladder contractions (SBCs) change to a low-amplitude, high-frequency pattern by about six weeks of age due to decreases in sensitivity to neurotransmitters, communication between smooth muscle cells via gap junctions, or the activity of pacemaker cells [49, 50]. BK channels were shown to play an important role in regulating SBCs in the neonatal mouse bladder by limiting excitability and contractility of bladder smooth muscle [50, 51]. Thus, an increase in the amplitude of SBCs were found in bladders of mice lacking the β1 subunit of the BK channel, and enhanced SBCs and increased sensitivity to electrical field stimulation and muscarinic agonists (carbachol) were observed in *Slo* ^*-/-*^ mice that lack the α subunit of the BK channels [51–53]. In bladders of *Slo* ^*-/-*^ mice excitability remains unchecked, causing detrusor muscle instability and urinary incontinence. This phenotype was similar in *Mras* ^*-/-*^ males and it remains to be determined whether M-Ras may affect BK function, or whether *Mras* ^*-/-*^ males may fail to undergo some aspects in the developmental changes from a newborn to an adult bladder. Alternatively, increased SBCs may develop secondary to bladder enlargement. Moreover, BK channels, activated initially through M3R-activated intracellular calcium release, were found to be inhibited by an M2R-mediated pathway, resulting in enhanced contractility of rat detrusors [54]. Thus, increased expression of M2R relative to M3R in *Mras* ^*-/-*^ males could contribute to carbachol-stimulated hypercontractility through this mechanism. These hypotheses will require further investigations.

In humans, non-obstructive chronic urinary retention (CUR) is typically caused by detrusor underactivity. The causes can be neurogenic (damage to nerves that innervate the bladder in conditions such as multiple sclerosis, spinal cord injury) or myogenic (damage to the smooth muscle of the bladder) [12, 15]. Another known cause of non-obstructive CUR is Fowler’s syndrome, a condition mainly affecting young women, where the urethral sphincter fails to relax [55], suggesting that in some cases Pudenal nerve function is important for voiding. In support of this, voiding was restored in rats with transsected Pudenal nerves by electrical stimulation of the afferent branches of the same nerve, thus providing sensory feedback [56]. Thus, it is possible that the signals from the Pudenal nerve to relax the urethral sphincter are below threshold in *Mras* ^*-/-*^ males under normal, non-stressed conditions, or that muscarinic receptor expression on the urethral sphincter may also be dysregulated. For example, if muscarinic (and purinergic) receptors are not expressed at adequate levels, or if ACh and/or ATP are not produced at adequate levels or at inappropriate times, signals from the Pudenal nerve to relax the urethral sphincter could be below threshold in *Mras* ^*-/-*^ males under normal, non-stressed conditions. That *Mras* ^*-/-*^ males were capable of voiding under unusual or stressed conditions (e.g. during a scent marking test) suggests that neurotransmitters were released in sufficient amounts under these circumstances; however, production may not be sufficient under resting or non-stressed conditions.

M-Ras participates in the reorganization of the actin cytoskeleton [57, 58] but its role in regulating smooth muscle function is presently unknown. Neuropilin 2 (Nrp2), a co-receptor with Plexin-A1 for Semaphorin 3F (Sema3F), is important for promoting detrusor smooth muscle relaxation through inhibition of RhoA and ROCK, and loss of Nrp2 enhances contractility in response to carbachol, K^+^ and other agonists by relieving this inhibition [59]. In neurons, the related receptor Plexin-B1, stimulated by Sema4D, is known to act as an inactivator (GTPase-activating protein; GAP) for M-Ras and R-Ras. Plexin-B1 signals via M-Ras and R-Ras lead to F-actin depolymerization and inhibit dendrite branching and outgrowth [58, 60]. It is conceivable that Nrp2 and Plexin-A1 via GAP activity of Plexin-A1 on M-Ras may contribute to the inhibition RhoA to promote F-actin depolymerization and detrusor relaxation. In support of this hypothesis, mice that have reduced levels of smooth muscle myosin isoforms SM1 and SM2 (SM2^+/-^ mice) exhibit a bladder phenotype that is strikingly similar to that of *Mras* ^*-/-*^ mice. About half of SM2^+/-^ males exhibit bladder distension at 18 months of age, and the detrusor is hypersensitive to both K^+^ depolarization and carbachol stimulation [61]. Thus, it is tempting to speculate that M-Ras might dampen smooth muscle contractility by negatively affecting smooth muscle actin (SMA) polymerization.

Other, less obvious causes could also contribute to a urinary phenotype in *Mras* ^*-/-*^ mice. For example, enlarged seminal vesicles due to bacterial disease led to urinary retention and frequency in a human patient [62]. Seminal vesicles were larger in 12 month-old *Mras* ^*-/-*^ males than in WT males (S4 Fig). It is presently unclear if this could contribute to retention in *Mras* ^*-/-*^ mice and/or if this might indicative of obstruction in the urethra. As a result of the deletion of *Mras* there could also be changes in the expression levels of other genes that could contribute or cause the urinary phenotype in *Mras* ^*-/-*^ males. Such potential compensatory mechanisms require further investigations.

In summary, we have uncovered a role for M-Ras in the urinary system in male mice. Mice developed chronic urinary retention with bladder enlargement and incontinence. We consider the dysregulation of muscarinic receptor expression a likely cause of retention, given the importance of M3R in the regulation male micturition and the failure to upregulate M3R by *Mras* ^*-/-*^ males to normal levels with age. *Mras* ^*-/-*^ males also exhibited increases in SBCs that likely contribute to bladder overactivity and incontinence. We propose that this may be facilitated by the relative high expression of M2R and subsequent inhibition of BK channels. Thus, the molecular details of the dysregulation of muscarinic receptors in *Mras* ^*-/-*^ males deserve further exploration.

## Supporting Information

S1 Fig Scoring system for the degree of ‘layering’ in detrusor smooth muscle. Normal detrusor shows compact organization of the smooth muscle tissue and receives a score of 0. With a medium amount of layering white spaces between smooth muscle layers are frequent; score = 1. Severe layering of smooth muscle showing a ‘bacon’-like morphology; score = 2.
(EPS)

S2 Fig Urine scent marking by WT and *Mras* ^*-/-*^ males and kidney weights. Four WT (A) and four *Mras* ^*-/-*^ (B) males were allowed to scent-mark new territory for 15 minutes. Urine spots were visualized by UV light. (C) Weights of both kidneys of WT and *Mras* ^*-/-*^ males at different ages. Error bars represent SD; WT three and six months, n = 14; WT 12 months, n = 17; *Mras* ^*-/-*^, three and 12 months, n = 15, *Mras* ^*-/-*^ six months, n = 19. Analysis by two-way ANOVA revealed a significant interaction between genotype and age (F_2,88_ = 6.413, *p* = 0.0025).
(EPS)

S3 Fig Detrusor contractility in WT and *Mras* ^*-/-*^ females. Bladder tissue from three month-old female mice near the apex (A) or the sphincter (B) was stimulated with increasing concentrations of the cholinergic agonist carbachol. Error bars represent SD, n = 3 for all groups. Two-way ANOVA: There was a significant effect of genotype for apex bladder tissue, F_1,28_ = 4.588, *p* = 0.0410, but not for tissue near the sphincter. Differences in amplitudes at specific carbachol concentrations were not significant in any of the samples tested.
(EPS)

S4 Fig Seminal vesicle weights of WT and *Mras* ^*-/-*^ males. Error bars represent SD, n = 14 for WT three and six months, n = 17 for WT 12 months, n = 15 for *Mras* ^*-/-*^ three and 12 months, n = 19 for *Mras* ^*-/-*^ six months. Two-way ANOVA: Although a significant interaction between genotype and age was detected, the difference in seminal vesicle weight was reported as significant at 12 months (F_1,88_ = 12.61, *p* = 0.0006; *p*<0.0001).
(EPS)

S5 Fig Transcript levels of receptors expressed by the urinary bladders of WT and *Mras* ^*-/-*^ mice. RNA expression levels of (A) muscarinic receptor 1 (M1R), (B) the β1 subunit of the large conductance calcium- and voltage-activated K^+^ channel (BK_Ca_β1), (C) purinergic receptor P2X1R, (D) fibroblast growth factor receptor 2 (FGFR2), (E) angiotensin II receptor 1a (AGTR1a), and (F) Neuropilin 2 (NRP2) are shown relative to expression levels of RP2. (A-F) Tissue near the apex of the bladder was used. Data is shown with SD; n = 3 for two month-old WT and *Mras* ^*-/-*^ females, n = 4 for two month-old WT and *Mras* ^*-/-*^ males and for six month-old females, n = 6 for six month-old WT males, n = 7 for six month-old *Mras* ^*-/-*^ males and females. Two-way ANOVA analyses did not reveal significant differences, with the following exceptions: There was a significant age-dependent decrease in M1R expression in female mice (F_1,13_ = 13.32, *p* = 0.0029) but no effect of genotype; P2X1R expression was significantly lower in six month-old *Mras* ^*-/-*^ males compared to WT males (F_1,17_ = 6.316, *p* = 0.0223; *p* = 0.0275); AGTR1a expression was significantly lower in two month-old *Mras* ^*-/-*^ vs. WT females (F_1,13_ = 16.07, *p* = 0.0015; *p* = 0.0185). (G) Primers used for qPCR in A-F.
(EPS)
